# *Ureaplasma parvum *infection alters filamin a dynamics in host cells

**DOI:** 10.1186/1471-2334-11-101

**Published:** 2011-04-20

**Authors:** Ayman B Allam, Sophie Alvarez, Mary B Brown, Leticia Reyes

**Affiliations:** 1 Department of Infectious Disease & Pathology, College of Veterinary Medicine, University of Florida, Gainesville, FL, USA; 2 Interdisciplinary Center for Biotechnology Research, University of Florida, Gainesville, FL 32611, USA; 3 Donald Danforth, Plant Science Center, 975 N. Warson Rd St. Louis, MO 63132, USA

## Abstract

**Background:**

*Ureaplasmas *are among the most common bacteria isolated from the human urogenital tract. *Ureaplasmas *can produce asymptomatic infections or disease characterized by an exaggerated inflammatory response. Most investigations have focused on elucidating the pathogenic potential of *Ureaplasma *species, but little attention has been paid to understanding the mechanisms by which these organisms are capable of establishing asymptomatic infection.

**Methods:**

We employed differential proteome profiling of bladder tissues from rats experimentally infected with *U. parvum *in order to identify host cell processes perturbed by colonization with the microbe. Tissues were grouped into four categories: sham inoculated controls, animals that spontaneously cleared infection, asymptomatic urinary tract infection (UTI), and complicated UTI. One protein that was perturbed by infection (filamin A) was used to further elucidate the mechanism of *U. parvum*-induced disruption in human benign prostate cells (BPH-1). BPH-1 cells were evaluated by confocal microscopy, immunoblotting and ELISA.

**Results:**

Bladder tissue from animals actively colonized with *U. parvum *displayed significant alterations in actin binding proteins (profilin 1, vinculin, α actinin, and filamin A) that regulate both actin polymerization and cell cytoskeletal function pertaining to focal adhesion formation and signal transduction (Fisher's exact test, P < 0.004; ANOVA, P < 0.02). This phenomenon was independent of clinical profile (asymptomatic vs. complicated UTI). We selected filamin A as a target for additional studies. In the BPH-1 model, we confirmed that *U. parvum *perturbed the regulation of filamin A. Specifically, infected BPH-1 cells exhibited a significant increase in filamin A phosphorylated at serine^2152 ^(P ≤ 0.01), which correlated with impaired proteolysis of the protein and its normal intracellular distribution.

**Conclusion:**

Filamin A dynamics were perturbed in both models of infection. Phosphorylation of filamin A occurs in response to various cell signaling cascades that regulate cell motility, differentiation, apoptosis and inflammation. Thus, this phenomenon may be a useful molecular marker for identifying the specific host cell pathways that are perturbed during *U. parvum *infection.

## Background

*Ureaplasma parvum *and *U. urealyticum *are among the most common bacteria isolated from the human urogenital tract [1-3]. Infection rates as high as 40 to 80% in women and up to 50% in men have been reported [3]. Most infections of the lower urogenital tract appear to be asymptomatic [1]. However, both species of *Ureaplasma *are also responsible for a variety of diseases such as chorioamnionitis, spontaneous abortion, premature birth, stillbirth, postpartum endometritis, neonatal neuropathies, and pneumonia with bronchopulmonary dysplasia [1,2,4,5]. Ureaplasmas are also implicated in a wide range of urinary tract diseases including urinary tract infection (UTI) [6], encrusted cystitis [7], urethritis [8], chronic prostatitis [9], and urolithiasis [10]. Most investigations have focused on elucidating the pathogenic potential of *Ureaplasma *species, but little attention has been paid to understanding the mechanisms by which these organisms are capable of establishing asymptomatic infection.

We previously developed an experimental model of UTI that has provided some insights into the host factors associated with asymptomatic infection and complicated disease [11-13]. Specifically, genetically inbred Fisher (F344) rats that were experimentally inoculated with *U*. *parvum *developed three clinical outcomes. Approximately one third of inoculated animals spontaneously cleared infection from the urinary tract by 2 weeks post inoculation. The animals that remained infected exhibited two distinct clinical profiles of UTI: asymptomatic infection or infection complicated by an exaggerated inflammatory response with bladder stone formation as sequela [11-13]. *U. parvum *organisms can be found colonizing the uroepithelial surface in both clinical profiles of UTI. However, in animals with complicated UTI, *U. parvum *can also be found within the submucosa of the bladder, which may be the driving force behind the persistent and exaggerated inflammatory response.

A notable feature in animals with asymptomatic UTI was the presence of quiescent uroepithelium despite the presence of *U. parvum*, which is in contrast to what occurs with UTI caused by other bacterial species [14,15]. Based on our observations, we postulated that ureaplasmas perturb uroepithelial function in a manner that interferes with innate immune defense and supports microbial colonization. In order to begin to identify the host cell processes that are perturbed by *Ureaplasma *during infection, we used differential proteomics to profile bladder tissues from F344 rats experimentally inoculated with *U. parvum*. Tissues from each clinical profile (sham inoculated control, culture negative animals, animals with asymptomatic UTI, and complicated UTI group) were analyzed in this study.

In this report we show that bladder tissue from animals actively colonized with *U. parvum *display significant alterations in actin binding proteins that regulate both actin polymerization and cell cytoskeletal function pertaining to focal adhesion formation and signal transduction. This phenomenon is independent of clinical profile (asymptomatic vs. complicated UTI). We selected the actin-binding protein filamin A as a target for additional studies based on proteome profiling results as well as its integral role in cell signaling events related to innate immunity [16,17]. We evaluated the impact of *U. parvum *infection on filamin A using the benign prostate hyperplastic (BPH-1) cell line as a model of infection. In the BPH-1 model, we confirmed that *U. parvum *perturbed the regulation of filamin A. Specifically, infected BPH-1 cells exhibited a significant increase in filamin A phosphorylated at serine^2152^, which correlated with impaired proteolysis of filamin A and its intracellular distribution.

## Methods

### Sample selection and protein extraction for rat bladder proteome studies

Rat bladder tissues from animals experimentally infected with a rat adapted strain of *U. parvum *were used for differential proteome profiling [12]. All procedures were performed in accordance with the University of Florida Institutional Animal Care and Use Committee. Briefly, animals were inoculated with sterile 10B broth (control group) or 10^9 ^CFU of *U. parvum*. Tissues were collected 2 weeks post-inoculation [12]. At time of tissue harvest, bladder from each animal was divided into 3 sections. One section was used for histopathology, another section was cultured for *U. parvum*, and the third section was flash frozen in liquid nitrogen and stored at -80°C for future analysis.

For proteome studies, tissues were grouped according to clinical profile. The negative group included animals inoculated with 10^9 ^CFU of *U. parvum *that were culture negative in the urinary tract at 2 weeks post inoculation (PI). Based on acute infection studies [11], 100% of animals inoculated with this dose were infected with *U. parvum *for at least 72 hours, therefore the negative group was composed of animals that spontaneously cleared infection. Animals within the UTI group were culture positive at time of necropsy, had minimal histologic changes in bladder tissue [12], low urine cytokine levels, and no evidence of struvite uroliths. Animals within the Struvite group were culture positive at time of necropsy, had extensive histologic changes in bladder tissue, were positive for struvites, and had marked elevations in urine pro-inflammatory cytokines. Animals sham-inoculated with sterile 10B broth served as uninfected controls. In order to minimize variability of *U. parvum *protein load between infected groups, only tissues that had similar log CFU (2.4 to 2.7) at time of necropsy were chosen for these experiments. Protein from tissues was extracted with Trizol (Invitrogen Corp., Carlsbad, CA) according to the manufacturer's protocol. Pelleted protein extracts were allowed to air dry and stored at -20°C before analysis.

### Quantitative proteomic analysis using peptide-labeling and offline 2D-LC-MS/MS

Three independent iTRAQ experiments were performed. Each experiment included one biological replicate from a control, negative, UTI, and struvite group. Sample processing, offline 2D-LC-MS/MS, protein identification and quantitation were performed as previously described [11]. Tandem mass spectra were extracted by Analyst (v 1.1.; Applied Biosystems/MDS Sciex). Concatenation of the forward and random sequences from the IPI rat database v 3.32 [18] were used for protein identification. Protein identification searches were performed using MS/MS data interpretation algorithms from Protein Pilot™ (Paragon™ algorithm, v 2.0, Applied Biosystems/MDS Sciex) [19] and Mascot (v 2.2, Matrix Science, London, UK). The confidence level for protein identification was set to 1.3 (95%). The false discovery rate for all iTRAQ™ experiments ranged from 0.0% to 0.93%. Protein ratios were generated with Pro Group™ algorithm and automatically corrected for bias. Protein quantification was performed with a minimum of three spectra that were present in all protein samples that were analyzed within the experiment. Only protein ratios with an error factor (EF) < 2 were retained for further analysis. EF is a measure of the variation among the different iTRAQ™ ratios (the greater the variation, the greater the uncertainty) and represents the 95% uncertainty range for a reported ratio. The calculated P-value obtained with the ProGroup™ algorithm is based on 95% confidence interval.

### Enrichment analysis of protein ratios that compared the Negative group to animals with active infection (UTI and Struvite groups)

Since our interest was to identify perturbations that were present in all animals with active UTI, regardless of the clinical profile, we only included protein ratios that exhibited the same response to infection in all infected groups. For example, if the protein ratio was decreased in both UTI and Struvite groups, then that protein was included. If the ratio was increased in the UTI group but decreased in the Struvite group, then that protein was considered to potentially be affected by the host inflammatory response and was excluded from the analysis. Proteins were grouped according to general biological functions as assigned in the Uniprot/Swissprot database. Protein ratios were considered significantly different if they had P values less than 0.05 as determined by the Pro Group™ algorithm; these proteins were assigned a binary value of 1. All others ratios were considered insignificant and were assigned a binary value of 0. Fisher exact test with Bonferroni correction for multiple comparisons was used to identify any biological function categories that were significantly over or underrepresented in animals with active UTI compared to animals that spontaneously cleared infection. Enrichment analysis was performed with JMP Genomics 3.0 software (SAS Institute Inc., Cary, NC).

### ANOVA and hierarchical clustering of rat bladder tissue proteome profiles

ANOVA was used to identify the proteome profiles that were common among animals with active UTI but significantly different from animals that cleared infection (Negative group). Protein ratios comparing Negative to control, UTI to control, and Struvite to control were generated with the Pro Group™ algorithm. Proteome datasets that contained only proteins with an EF < 2 and that also were identified in all three independent iTRAQ experiments were analyzed with JMP Genomics 3.0 software (SAS Institute Inc., Cary, NC). The quality of the data was assessed by distribution analysis, box plots and kernel density estimates and standardized prior to ANOVA (row by row modeling). ANOVA was performed with a false discovery rate set at α = 0.05. The least squares means of proteins that significantly differed among groups (P < 0.03) were then clustered by the method of Ward (protein to protein within group, as well as group to group).

### Infection studies with benign prostate hyperplastic cells (BPH-1)

BPH-1 cells were a gift from Dr. Charles Rosser (MD Anderson Cancer Center, Orlando, FL). For all experiments BPH-1 cells were cultured at 37°C in 5% CO_2 _in complete RPMI containing 10% fetal calf serum. For each experiment, the number of viable cells was determined by trypan blue staining. Cell numbers were adjusted to a concentration of 10^7 ^cells per ml and plated in 6 well plates (Corning Inc., Lowell, MA) or Lab-Tek II 8 well glass slide chambers (Nuncbrand, Rochester, NY). After 24 hours, the cell culture medium was changed with fresh complete RPMI and cells were checked for 50 to 60% confluence prior to infection with *U. parvum*. Each experiment contained three biological replicates within each treatment group, and each experiment was repeated at least twice.

For supernatant experiments, 10 ml of RPMI medium was harvested from BPH-1 cells that were exposed to sterile 10B broth or 10^9 ^CFU of *U. parvum *for 24 hours. Harvested cell supernatants were first clarified by centrifugation at 15,000 × g at 4 C for 10 minutes to remove any cell debris. The clarified supernatant was then filtered through sterile 0.1 μm syringe filters to remove any bacteria. BPH-1 cell cultures were inoculated with processed supernatants and maintained at 37°C and 5% CO_2 _for 72 hours before harvesting.

### Preparation and culture of *U. parvum*

For all BPH-1 cell infection experiments, our rat adapted strain of *U. parvum *[13] was grown to mid log phase (approximately 14 hours), which was confirmed by optical density reading obtained at 550 nm. The log CFU of each inoculum was also confirmed by culture on A8 agar as previously described [13].

### Immunocytologic assays

Anti-*U. parvum *rabbit polyclonal antibody (a gift from Dr. Janet Robertson, Medical Microbiology and Immunology, University of Alberta) was used to detect bacteria in BPH-1 cell cultures. Rabbit monoclonal antibody clone EP2405Y (Epitomics, Burlingame, CA) that recognizes the C terminal region of filamin A was used to detect intact, and cleaved forms of the molecule. Mouse monoclonal anti-filamin 1 (clone SPM182 from Santa Cruz Biotechnology, Inc, Santa Cruz, CA) was used to detect the intact form of the whole molecule. Rabbit monoclonal antibody clone EP2310AY was used to detect Filamin A phosphorylated at serine^2152 ^(Abcam Inc., Cambridge, MA). Rabbit and mouse IgG isotype controls were used to assess non-specific binding of primary antibodies (Thermo Scientific, Fremont, CA). For detection purposes, secondary antibodies were Alexa Fluor-488 goat anti-mouse IgG and ALEXA Fluor-594 goat anti-rabbit IgG (Invitrogen, Corp., Carlsbad, CA). Nuclei were stained with DAPI and polymerized actin was stained with Phalloidin labeled with Alexa Fluor-488 (Invitrogen, Corp., Carlsbad, CA).

Cells grown on sterile glass slides were fixed for 3 minutes with 3.7% formaldehyde in phosphate buffered saline (PBS), and processed for immunofluorescent staining as previously described [11]. Images were captured with Olympus IX81-DSU Spinning Disk confocal Microscope using Slidebook software (Olympus, Center Valley, PA).

For determination of *U. parvum *infection rates, foci that demonstrated colocalization of DAPI with anti-*U parvum *antibody were counted as positive. For quantification of cells with normal intracellular filamin A distribution, cells that exhibited strong intranuclear staining of filamin A were counted as positive. For all studies, a minimum of five biological replicates were evaluated and at least 200 cells per sample were counted.

### Preparation of whole cell lysates for ELISA

Adherent cells were gently washed twice with sterile PBS. Cells were then lysed with 1 ml of ice cold lysis buffer [50 mM Tris, pH 7.5, 0.15 M NaCl, 2 mM EDTA, 1 mM EGTA, 1% Triton -X] supplemented with HALT protease inhibitor and HALT phosphatase inhibitor (PIERCE Chemicals, Rockford, IL.). Cell suspensions were transferred to sterile tubes and sonicated with three 10 second pulses (Sonic Dismembrator model 500, Fisher Scientific, Pittsburgh, PA). Disrupted cell suspensions were divided into 200 μl aliquots and stored at -80°C.

### Detection of total and phosphorylated filamin A by ELISA

OptEIA ELISA reagent kit B (BD Biosciences, San Diego, CA) was used to perform the assay as previously described [11]. Reagents used in this study were mouse monoclonal anti-filamin 1 (clone SPM182 from Santa Cruz Biotechnology, Inc, Santa Cruz, CA) for capture, c terminal filamin A rabbit monoclonal (Epitomics, Burlingame, CA) for detection of total filamin A, and phospho^S2152 ^filamin A rabbit polyclonal antibody (ab75978 from Abcam Inc., Cambridge, MA) to detect the phosphorylated protein. For normalization purposes, the total protein concentration of each sample was determined by micro BCA protein assay (Pierce Chemicals, Rockwood, MD). Absorbance values (ABS) obtained by ELISA were divided by their total protein concentration so that values are reported as ABS/mg of total protein.

### Western blot analysis

Filamin A was detected with rabbit monoclonal anti-C terminal antibody (Epitomics, Burlingame, CA). Calpastatin, calpain, and GAPDH were detected with rabbit polyclonal antibodies (Abcam, Cambridge, MA). GAPDH was used as a loading control. Nuclear and cytoplasmic fractions were prepared with NE-PER nuclear and cytoplasmic extraction kit supplemented with HALT™ protease inhibitor and HALT™ phosphatase inhibitor (Thermo Scientific, Rockford, IL).

Cell extracts were loaded onto a NuPAGE^® ^10% Bis-Tris gel (Invitrogen, Carlsbad, CA) and subjected to 105 V for 1.5 hours in MOPS-SDS running buffer. Protein transfer to nitrocellulose membrane filter paper, 0.45 μm pore size (Invitrogen, Carlsbad, CA), was performed at 30 V for 1.5 hours in transfer buffer [Bicine 25 mM, Bis-Tris 25 mM, EDTA 1 mM, and 10% methanol]. Detection was performed with Super Signal^® ^West Pico Complete Rabbit IgG Detection Kit (Thermo Scientific, Rockford, IL) according to manufacturer's instructions. Chemiluminescence was detected with ChemiDoc™ Imaging system and densitometry of imaged bands was performed with Quantity One v 4.6.9 software (Biorad Laboratories Inc. Hercules CA).

### Statistical data analysis of BPH-1 cell experiments

Data from multiple experiments were grouped together in order to make statistical analysis possible. Data were analyzed by one-way ANOVA when more than two groups were included in the analysis. Fisher's Protected Least Significant Difference (PLSD) test was used when ANOVA indicated a significant difference among group means. Unpaired student's t test was used for comparisons that were limited to two groups. For all analyses, a probability of P ≤ 0.05 was considered significant.

## Results

### Proteome profiling of F344 rat bladder tissues

**Figure 1:**
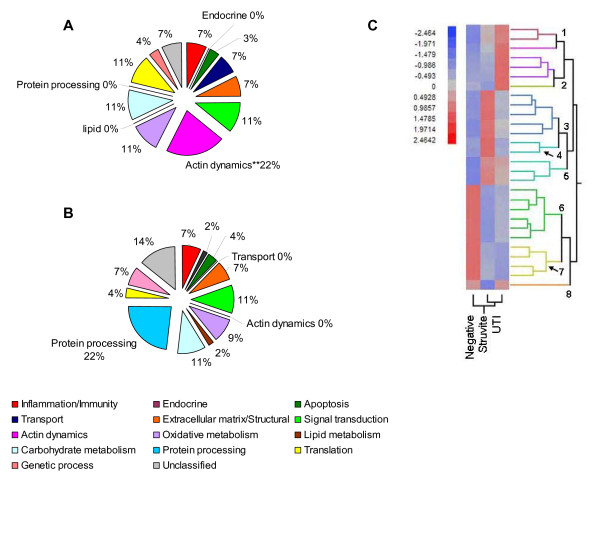
**Proteome profiling of F344 rat tissues inoculated with sterile broth or *U. parvum***. Panels A and B represent the percent of proteins assigned to each biological function group. Gene ontology designations were obtained from the Uniprot/Swissprot Database. Protein ratios of each specific protein are from UTI and Struvite groups divided by the Negative group (n = 3). Graph A shows the distribution of protein ratios that exhibited a significant difference by Pro Group™ algorithm (P < 0.05). **Biological function categories that were determined to be significantly different by enrichment analysis. Graph B shows the distribution of protein ratios that were not significantly different between Negative group and groups that were culture positive for *U. parvum *(UTI and Struvite). Panel C is a hierarchical cluster of standardized least squares means that were significantly different (P < 0.02) among Negative, Struvite, and UTI groups. Ratios generated with the ProGroup™ algorithm were analyzed by ANOVA (n = 3) with a false discovery rate α = 0.05.

Two approaches, enrichment analysis and ANOVA, were used to identify proteins that displayed the same response to *U. parvum *infection regardless of the clinical profile (UTI or Struvite). In the first approach, enrichment analysis was performed on protein ratios that were generated by comparing the Negative group to animals that were still actively infected with *U*. *parvum *(both UTI and Struvite groups). Only proteins that were identified in all three independent iTRAQ experiments and that also exhibited a common effect among animals actively infected with *U. parvum *were used for this analysis. Using these criteria, 28 of 84 proteins exhibited both a significant (P < 0.05) and a common effect with *U. parvum *colonization (listed in Additional file 1, Table S1). The distribution of these proteins according to their biological function is summarized in Figure 1a and 1b. Enrichment analysis revealed that animals colonized with *U. parvum *exhibited a significant change in proteins that regulate actin polymerization (P < 0.004, with Bonferroni correction). These actin-regulating proteins were profilin 1, filamin A, α actinin, vinculin, spectrin and talin. With the exception of profilin 1, all actin binding proteins were significantly lower in animals colonized with *U. parvum *(Additional File 1, Table S1).

In the second analysis, we compared the proteome profile of animals inoculated with *U*. *parvum *to the proteome profile from sham inoculated controls. All protein ratios (Negative/control, UTI/control, and Struvite/control) that had an EF < 2 and that were identified in all three iTRAQ experiments were then analyzed by ANOVA. Twenty-eight of 315 proteins displayed a significantly different pattern among Negative, UTI, and Struvite groups (P < 0.03). These proteins are listed in Additional file 1, Table S2. The least squared means of the 28 proteins were clustered and are presented in Figure 1C. Protein clusters 5, 6, and 7 showed a similar pattern in UTI and Struvite groups which was markedly different from the Negative group. Cluster 5 contained apolipoprotein A-I precursor, peptidyl-prolyl cis-trans isomerase B precursor, and calmodulin. Cluster 6 contained isocitrate dehydrogenase [NADP], EH-domain containing 2 protein, α-enolase, peroxiredoxin-2, creatine kinase B-type, and complement C3 precursor. Cluster 7 contained elongation factor 1α, α-actinin, vinculin, and filamin A.

Table 1 lists the 8 proteins found to be significantly affected by *U. parvum *as detected by both enrichment analysis and ANOVA. Four of these proteins (profilin 1, α actinin, vinculin, and filamin A) are involved in the regulation of actin polymerization. With the exception of profilin 1, these actin binding proteins were significantly decreased in animals with active infection.

**Table 1:** List of proteins found to be perturbed in animals with active *U. parvum *infection^a)^.

Accession^b)^	Protein name	**Gene Ontology Biological Process^c)^**	Effect^d)^
IPI00194097.5	Gc Vitamin D-binding protein	transports vitamin D and its metabolites	**↑**
IPI00193485.2	Isocitrate dehydrogenase [NADP]	carbohydrate metabolism	**↓**
IPI00767147.1	Similar to Alpha-enolase	carbohydrate metabolism	**↓**
IPI00195372.1	Elongation factor 1-alpha 1	protein biosynthesis/translational elongation	**↓**
IPI00231358.6	Profilin - 1	actin binding	**↑**
IPI00454431.1	Brain-specific alpha actinin 1	actin binding	**↓**
IPI00365286.3	Similar to Vinculin	cell motility/cell adhesion/lamellipodium biogenesis	**↓**
IPI00409539.3	Similar to Filamin-A	actin filament binding/actin cytoskeleton reorganization/glycoprotein binding/cytoplasmic sequestering of protein/regulation of transcription factor/regulation of I-kappa β kinase/NF-κβ cascade	**↓**

a) Proteins found to be significantly altered by both enrichment analysis (Figure 1A and B) and ANOVA (Figure 1b).

b) Accession numbers refer to the International Protein Database, http://www.ebi.ac.uk/IPI/IPIhelp.html.

c) Gene ontology data was obtained from the Panther database http://www.pantherdb.org and the UniprotKB/Swiss-Prot Database http://www.ebi.ac.uk/uniprot.

d) Effect refers to the protein ratio that was generated by the ProGroup™ algorithm, which compared actively infected animals (UTI and Struvite groups) to Negative group or Control group. Actual protein ratios are listed in Additional file 1, tables S1 and S2.

Because filamin A is one of the better characterized proteins and is involved in regulating both signal transduction and gene expression [16,17], we chose to focus our attention on this key protein. We evaluated the distribution of filamin A in rat bladder tissues by immunofluorescent staining with the monoclonal antibody specific for intact filamin A (see Additional file 2). There was no appreciable difference in the overall intensity or distribution of filamin A staining in the tissues of uninfected and infected animals. Therefore, in order to further characterize the effect of *U. parvum *colonization on filamin A dynamics of host cells, it was necessary to expand our studies to a cell culture system.

### Infection of human BPH-1 cells with *U. parvum*

We established a model of infection using the BPH-1 immortalized, differentiated epithelial cell line [20,21]. In addition to being amenable to chronic infection with mycoplasmas [22], prostate cells exhibit a distinct intracellular distribution of filamin A that facilitates evaluating its dynamics [22].

In preliminary studies, the frequency of *U. parvum *colonization of BPH-1 cells was evaluated by confocal microscopy at 24, 48, and 72 hours. In all experiments, *U. parvum *organisms were consistently found on the host cell membrane (see Additional file 3). *U*. *parvum *colonization of BPH-1 cells was detected by both DAPI staining and *U. parvum *specific antibody labeling. At 24 hours, *U. parvum *could be detected on 97 ± 3.5% (mean ± SD, n = 4) of cells that were evaluated in two separate experiments. At 48 hours, colonization rates were reduced to 60.5 ± 15%. By 72 hours, 51 ± 7.8% of cells were colonized. We selected the 72 hour post inoculation time point for all additional experiments since colonization rates appeared to stabilize by that time point.

### Intracellular distribution of filamin A in BPH-1 cells

**Figure 2:**
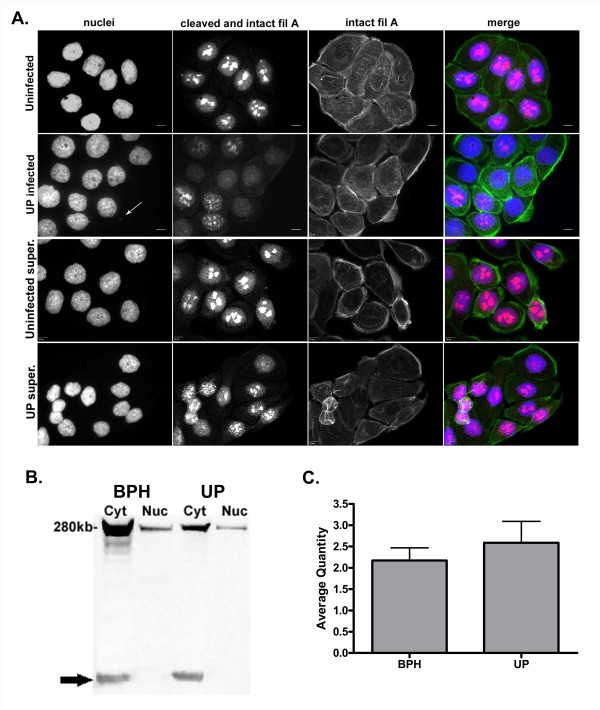
**Intracellular distribution intact and cleaved filamin A in BPH-1 cells**. Cells were exposed to sterile 10B broth, 10^9 ^CFU of *U. parvum *(UP), or cell culture supernatant (super) for 72 hours before examination by confocal microscopy (A), Western blot (B) and densitometry (C). Confocal images were taken at 600× magnification and the scale bar is equal to 10 μm. Cleaved and intact filamin A (Fil A) were stained with rabbit- anti C terminal filamin A (red). Intact Fil A was stained with mouse anti-filamin 1 (green). BPH-1 nuclei and *U. parvum *(white arrow) were identified with DAPI stain (blue). Western blot analysis for the detection of cleaved filamin A was performed on cytosolic (cyt) and nuclear (nuc) fractions from uninfected (BPH) and infected (UP) cells. The black arrow is delineating GAPDH, which was used as a loading control and a confirmation that the nuclear fraction was not contaminated with cytosolic proteins. Quantitation of intact filamin A was performed by densitometry of the cytosolic fractions of uninfected and *U. parvum *infected cells. The average quantity within each blot was normalized by dividing the average quantity of filamin A protein band by the average quantity of the GAPDH band. Values represent the mean ± SD of 3 replicates from 3 independent experiments.

We evaluated the intracellular distribution of filamin A in uninfected and infected BPH-1 cells by confocal microscopy and immunoblotting. Uninfected cells showed a punctate pattern of filamin A within the nucleus (see Figure 2a) that was seen only with the antibody that recognized the cleaved form of filamin A. When a monoclonal antibody that recognized only intact filamin A was used, nuclear filamin A appeared to be present in a striated form that resembled actin stress fibers. This was confirmed with co-localization studies of filamin A with polymerized actin as shown in Additional file 3. This intracellular distribution of filamin A was consistent with previous reports in normal prostate cells [23,24]. Interestingly, a significant proportion of *U. parvum *infected BPH-1 cells exhibited a marked reduction in the punctate nuclear staining of filamin A coupled with a concurrent increased of filamin A in the cytosol (P < 0.0001). Specifically, 68 ± 10% of *U. parvum *infected cells as compared with 14.4 ± 4% of uninfected cells showed this abnormal phenotype.

In order to determine if the abnormal filamin A phenotype could be the result of a paracrine mediated host response to infection, we exposed BPH-1 cells to culture supernatants obtained from sham inoculated or *U. parvum *infected cultures. Supernatants were harvested from BPH-1 cell cultures at 24 hours post inoculation with *U. parvum*. The 24 hour time point was chosen because cell culture supernatants obtained at this post inoculation time point contain the highest cytokine/chemokine concentrations (unpublished studies in our laboratory). Moreover, we wanted to minimize any potential confounding factors that could result from incubating cells with nutrient depleted medium, which could occur if a longer post inoculation time point was selected. As shown in the two bottom panels in Figure 2a, supernatant treated cultures did not display an abnormal filamin A phenotype as was detected in *U. parvum *infected cultures.

We evaluated both nuclear and cytosolic fractions of cell lysates by Western blot with the antibody that recognizes the C-terminal portion of filamin A. As shown in Figure 2b, there was a marked reduction in the detection of filamin A fragments in lysates from *U. parvum *infected cultures. Densitometry of intact filamin A in the cytosolic fraction of cell lysates was also performed (Figure 2c). For this analysis, ratios were generated by dividing the average quantity of intact filamin A by the average quantity of GAPDH that was used as a loading control. As shown in Figure 2c, the relative amount of intact filamin A was greater in the cytosolic fraction of *U. parvum *infected cells. The total amount of filamin A was also measured by ELISA, and no significant difference in the total amount of filamin A among infected and uninfected BPH-1 cells was detected (data not shown).

### Detection of phosphorylated filamin A in BPH-1 cells

**Figure 3:**
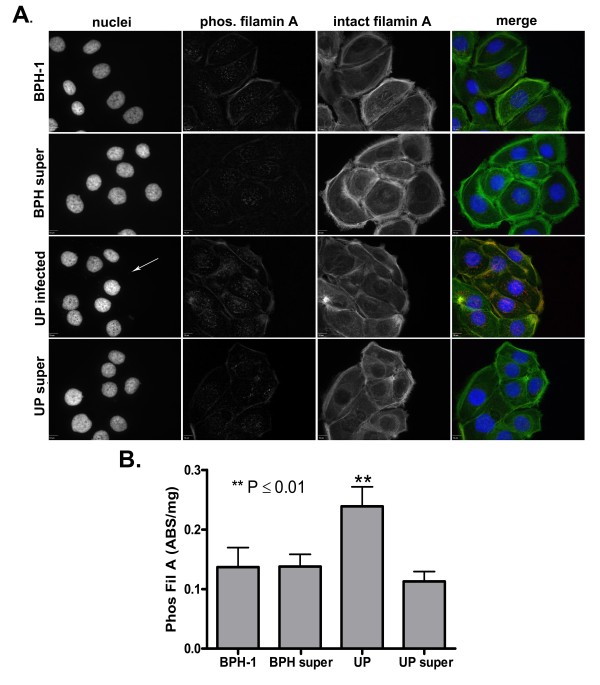
**Intracellular distribution and quantification of phosphorylated filamin A in uninfected, *U. parvum *infected, and supernatant treated BPH-1 cells**. Cells were exposed to sterile 10B broth, 10^9 ^CFU of *U. parvum*, or cell culture supernatant (super) for 72 hours before examination by confocal microscopy (A), or ELISA (B). Phosphorylated filamin A (red) was detected with a rabbit monoclonal antibody (EP2310AY). Intact filamin A (green) was detected with mouse anti-filamin 1. BPH-1 nuclei and *U. parvum *(white arrow) were identified with DAPI stain (blue). All images were taken at 600× magnification and the scale bar is equal to 10 μm. ELISA for phosphorylated filamin A was performed on whole cell lysates. Absorbance values were divided by the total mg protein determined by BCA assay. Values represent the mean ± SD (n = 5) of phosphorylated filamin A in uninfected (BPH-1) and infected (UP), uninfected supernatant treated (BPH super), and infected supernatant treated (UP super) cells. **P Value Was obtained by Fishers PLSD.

In prostate cells, a dominant pathway of filamin A regulation involves cleavage of the protein by calpain [23,24]. Calpain mediated cleavage of filamin A can be reduced by phosphorylation of the protein at serine^2152^. Therefore, we assessed the degree of filamin A phosphorylation at serine^2152 ^by immunofluorescent microscopy (Figure 3A) and ELISA (Figure 3B). Both detection methods confirmed that only cells infected with *U. parvum *displayed a significant increase in phosphorylated filamin A.

### Detection of calpastatin and calpain in BPH-1 cells

**Figure 4:**
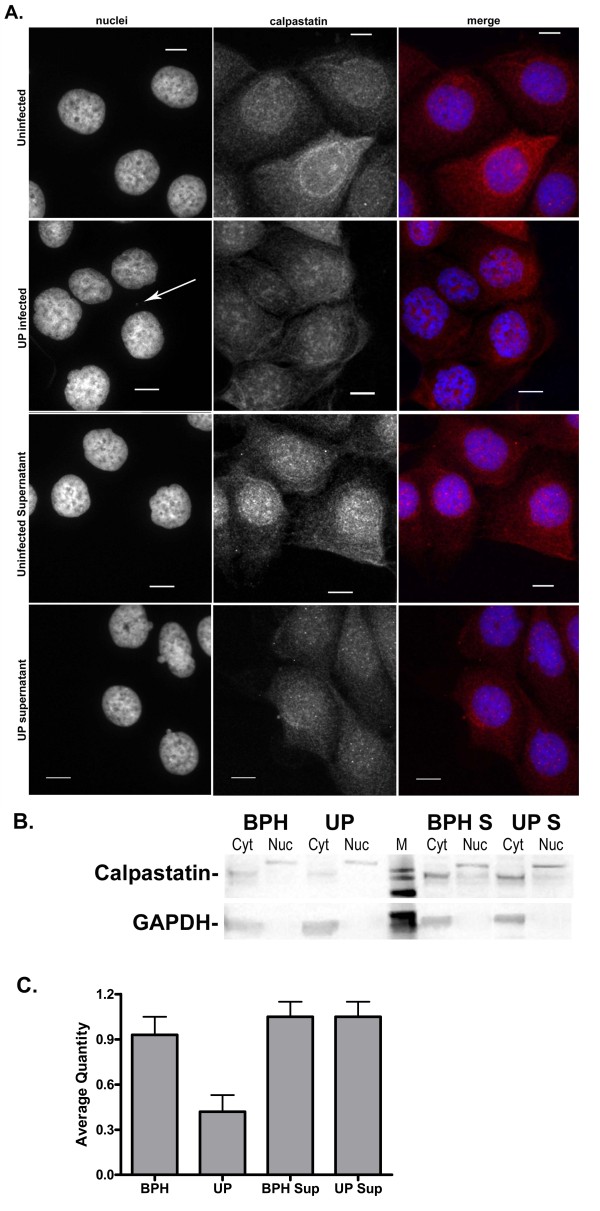
**Intracellular distribution and quantification of calpastatin in uninfected, *U. parvum *infected, and supernatant treated BPH-1 cells**. Cells were exposed to sterile 10B broth, 10^9 ^CFU of *U. parvum*, or cell culture supernatant for 72 hours before examination by confocal microscopy (A), Western blot (B) and densitometry (C). Confocal images were taken at 600× magnification and the scale bar is equal to 10 μm. Calpastatin was detected with rabbit polyclonal antibody (red). BPH-1 nuclei and *U. parvum *(white arrow) were identified with DAPI stain (blue). Western blot analysis for the detection of calpastatin was performed on cytosolic (cyt) and nuclear (nuc) fractions from uninfected cells (BPH), infected (UP), uninfected supernatant treated (BPH S) and infected supernatant treated (UP S) cells. M equals molecular weight marker. GAPDH was used as a loading control and a confirmation that the nuclear fraction was not contaminated with cytosolic proteins. Quantitation of calpastatin in cytosolic fractions was performed by densitometry. The average quantity within each blot was normalized by dividing the average quantity of calpastatin protein band by the average quantity of the GAPDH band within each blot. Values represent the mean ± SD of 2 biological replicates from 2 independent experiments.

Recent studies have shown that infection of host cells with *Mycoplasma hyorhinis *caused inhibition of calpain activity through upregulation of its inhibitor, calpastatin [25]. Therefore, we also evaluated the effect of *U. parvum *infection on the intracellular distribution and the relative concentrations of calpain and calpastatin in BPH-1 cells. We did not detect a difference in the intracellular distribution of calpain among uninfected BPH-1 cells, *U. parvum *infected cells, and cells incubated with supernatants by confocal microscopy (data not shown). We also did not observe any appreciable differences in the amount of calpain present within the cytosolic and nuclear fractions of these cells by Western blot (data not shown). However, we did observe differences in both the intracellular distribution of calpastatin and its relative concentration among the groups. Specifically, *U. parvum *infected cells exhibited large aggregates of calpastatin within the nucleus, and these aggregates were more prominent than what was observed in the other groups (Figure 4A). Moreover, Western blot showed that calpastatin was reduced in the cytosolic fraction of *U. parvum *infected cells (Figure 4B), which was confirmed by densitometry (Figure 4C.)

## Discussion

In our previous study, we found that colonization of the mucosal surface of the bladder was a common feature in inbred F344 rats with either asymptomatic or complicated UTI [12]. Based on these findings, we postulated that *U. parvum *may be causing a disruption in epithelial host cell function in a manner that supports microbial colonization. In order to identify host cell proteins that may be perturbed by *U. parvum *colonization, we used differential proteome profiling to compare animals with active UTI to animals that spontaneously cleared infection or with sham inoculated controls. We used both enrichment analysis and ANOVA to identify correlations between colonization with *U. parvum *and perturbations in functional protein categories or protein networks. Both methods revealed that animals colonized with *U. parvum *exhibited significant perturbations in proteins that normally regulate actin polymerization during cell attachment, motility and signal transduction events [26-31]. Specifically, the concentration of profilin 1, α actinin, vinculin, and filamin A were found to be significantly altered by infection.

We chose to focus our additional experiments on filamin A because its regulation has been studied more extensively than that of profilin 1, α actinin, and vinculin [16,17,23,24,26]. This protein is also involved in the regulation of various cell signaling events including pathways that are important in both innate and adaptive immunity [32-35]. According to the ProGroup™ analysis, filamin A was significantly reduced in animals actively colonized with *U. parvum*. However, immunofluorescent detection of filamin A in rat bladder tissues did not support the proteome data. Thus, the changes that we observed in infected tissues may be a reflection of post-translational events such as proteolysis or chemical modifications that affect protein solubility and recovery during protein extraction [27,28]. Therefore, it was necessary to develop an *in vitro *model of infection that could be used to elucidate these mechanisms. BPH-1 cells were used as our model system because they are a differentiated, immortalized epithelial cell line of the urogenital tract [20,21] that is amenable to long-term colonization with *Mollicutes *[22]. Moreover, prostate cells display a distinct intracellular distribution of filamin A that we were able to exploit in our studies [23,24]. Specifically, filamin A cleavage mediated by calpain is a dominant pathway in prostate cells. Once filamin A is cleaved, the C - terminal fragments of the protein translocate to the nucleus [23,24]. We observed C - terminal fragments of filamin A within the nucleus of uninfected cells by confocal microscopy. We also observed these fragments within the cytosolic fraction of BPH-1 cells by Western blot. However, we did not detect these fragments within the nuclear fraction of these cells by this method, which is most likely a reflection of the detection limits of the assay. Despite this limitation, both confocal microscopy and Western blot identified a disruption in the normal process of filamin A cleavage in *U. parvum *infected cells. This effect appears to be due to the actual presence of the microbe since it was not observed in cells exposed to cell culture supernatants from infected cultures.

Two principle mechanisms can account for the decreased proteolysis of filamin A. The protein itself becomes resistant to calpain mediated cleavage when it is phosphorylated at serine^2152 ^[36,37]. The second mechanism involves direct inhibition of calpain by its natural inhibitor calpastatin [38,39]. Changes in intracellular concentrations of calpain and calpastatin have been shown to correlate with changes in filamin A cleavage [40]. The results of our studies suggest that phosphorylation of filamin A is the most likely mechanism for *U. parvum *mediated disruption in this system. *U. parvum *infected cells had significantly greater amounts of filamin A phosphorylated at serine^2152 ^than did the other groups (uninfected cells or cells incubated with supernatants). *U. parvum *infection did not appear to affect the intracellular distribution of calpain or its overall concentration as detected by Western blot. Furthermore, the changes we observed in calpastatin dynamics of *U. parvum *infected cells could actually reduce the ability of calapstatin to inhibit calpain [41,42].

Phosphorylation of filamin A may be a downstream effect of signal transduction that is initiated at the host cell membrane/microbe interface. Both cAMP-dependent kinase (PKA) [37] and ribosomal S6 kinase (RSK) [43] have been reported to endogenously phosphorylate filamin A at serine^2152^. Therefore, both kinases are potential upstream effectors of *U. parvum *mediated effects on filamin A. However, our results suggest the cAMP pathway may be the more likely target of *U. parvum *infection. For example, the perturbation of calpastatin dynamics observed in infected BPH-1 cells can occur with increased intracellular cAMP and activation of PKA [41]. The changes in vinculin, α actinin, α enolase, and elongation factor 1α that were detected by differential proteome profiling of bladder tissues can also be the result of increased intracellular cAMP [44-47]. Downstream effectors of cAMP such as EPAC (exchange protein directly activated by cAMP, also known as cAMP-GEF) and PKA have been shown to modulate inflammation and tissue proliferation [48,49]. Thus, elucidating the upstream components of filamin A phosphorylation may provide new mechanistic insights into the mechanisms of ureaplasmal asymptomatic infection and disease.

The effects we observed in *U. parvum *infected BPH-1 cells can provide an explanation for the reduction of filamin A that was detected in the iTRAQ experiments. The phosphorylation of filamin A that was noted in *U. parvum *infected cells correlated with its redistribution into less soluble compartments of BPH-1 cells (cell membrane and the cell cytoskeleton). Since less soluble proteins can be lost by Trizol extraction methods, they can be underrepresented within the fraction and measured as a decrease by iTRAQ™ analysis. Unfortunately, none of the available antibodies specific for phosphorylated filamin A at serine^2152 ^worked in rat bladder tissues so we could not determine if this also occurred in infected animals. Despite these limitations, both models of infection displayed a perturbation of filamin A dynamics, which may serve as a viable molecular marker for delineating the host cell signal transduction pathways that are affected by *U. parvum *infection.

## Conclusions

Proteome profiling of rat bladder tissues identified a significant perturbation in host cell filamin A during colonization with *U. parvum*. *In vitro *infection studies with BPH-1 cells confirmed that *U. parvum *colonization interfered with the normal distribution of intracellular filamin A by inducing phosphorylation of the protein at serine^2152^. Phosphorylation of filamin A occurs in response to various cell signaling cascades that regulate cell motility, differentiation, apoptosis and inflammation, which may be relevant to ureaplasmal disease pathogenesis. Thus, this phenomenon may be a useful molecular marker for identifying the specific host cell signaling pathways perturbed during *U. parvum *infection.

## Competing interests

The authors declare that they have no competing interests.

## Authors' contributions

AA executed cell culture experiments and contributed to manuscript preparation. SA designed and executed proteome studies, and assisted in manuscript preparation. MBB participated in the design and coordination of the study and assisted in manuscript preparation. LR designed experiments, executed animal infection studies, assisted in cell culture experiments, data analysis and manuscript preparation. All authors concur with the final version of the manuscript.

## Pre-publication history

The pre-publication history for this paper can be accessed here:

http://www.biomedcentral.com/1471-2334/11/101/prepub

## Supplementary Material

Additional file 1 **Table S1 - Rat bladder proteins that were significantly altered by active infection with *U. parvum *as determined with the Pro Group™ algorithm**. Table S1 contains the list of protein ratios that significantly differed among rats inoculated with *U. parvum *(active infection versus cleared infection). Results are presented as the mean ± SD of three biological replicates obtained from three independent iTRAQ™ experiments. Ratios were generated by dividing the spectral intensity value in UTI and Struvite groups by the spectral intensity for a specific peptide in the Negative group. Only protein ratios that showed a similar response in both UTI and Struvite groups and were significantly different from the Negative group (P < 0.05) are listed. Proteins were grouped according to their assigned biological function. **Table S2 - Rat bladder tissue proteome profiles that were significantly different among rats inoculated with *U. parvum *as determined by ANOVA**. Table S2 contains the list of protein ratios obtained with the ProGroup™ algorithm (mean ± SD) that were significantly different among rats inoculated with *U. parvum *as determined by ANOVA. Data was obtained from three biological replicates from three independent iTRAQ™ experiments. Protein ratios were generated by dividing the spectral intensity of the protein in a *U. parvum *inoculated group (Negative, UTI, or Struvite) with the spectral intensity in the sham inoculated control using the ProGroup™ algorithm. Proteins that showed a significantly different profile (P < 0.03) are listed. Proteins are grouped according to the cluster pattern shown in Figure 1C that was obtained by the method of Ward. The biological function of each protein was obtained from the Panther database http://www.pantherdb.org or the Rat Genome Database http://rgd.mcw.edu/wg. Click here for file

Additional file 2 **Immunohistochemical detection of intact filamin A in the bladder tissue of F344 rats inoculated with sterile 10B broth or 10^9 ^CFU of *U. parvum***. Representative bladder tissue sections from isotype primary antibody control (A), sham inoculated control (B), asymptomatic UTI (C), and struvite (D) groups demonstrating the distribution of intact filamin A (green). Nuclei were stained with DAPI (blue). Images are 600× magnification, L = bladder lumen, SM = submucosa, and arrows are pointing to uroepithelium. Click here for file

Additional file 3 **Colocalization of filamin A with polymerized actin in uninfected and *U. parvum *infected BPH-1 cells**. Representative images of cells examined 72 hours after inoculation with sterile 10B broth or 10^9 ^CFU of *U. parvum*. Nuclei were stained with DAPI (blue), white arrows are pointing to *U. parvum *colonies that were detected with DAPI staining. Filamin A (Fil A) was stained with rabbit- anti C terminal filamin A (red). Polymerized actin was stained with phalloidin Alexa-448 (green). All images were taken at 400× magnification and the scale bar is equal to 10 μm. Click here for file
