# Supplementary figures and images for: Ureaplasma parvum infection alters filamin a dynamics in host cells
Source: BMC Infect Dis. 2011 Apr 20;11:101. doi: 10.1186/1471-2334-11-101 (PMC3107797; doi:10.1186/1471-2334-11-101)

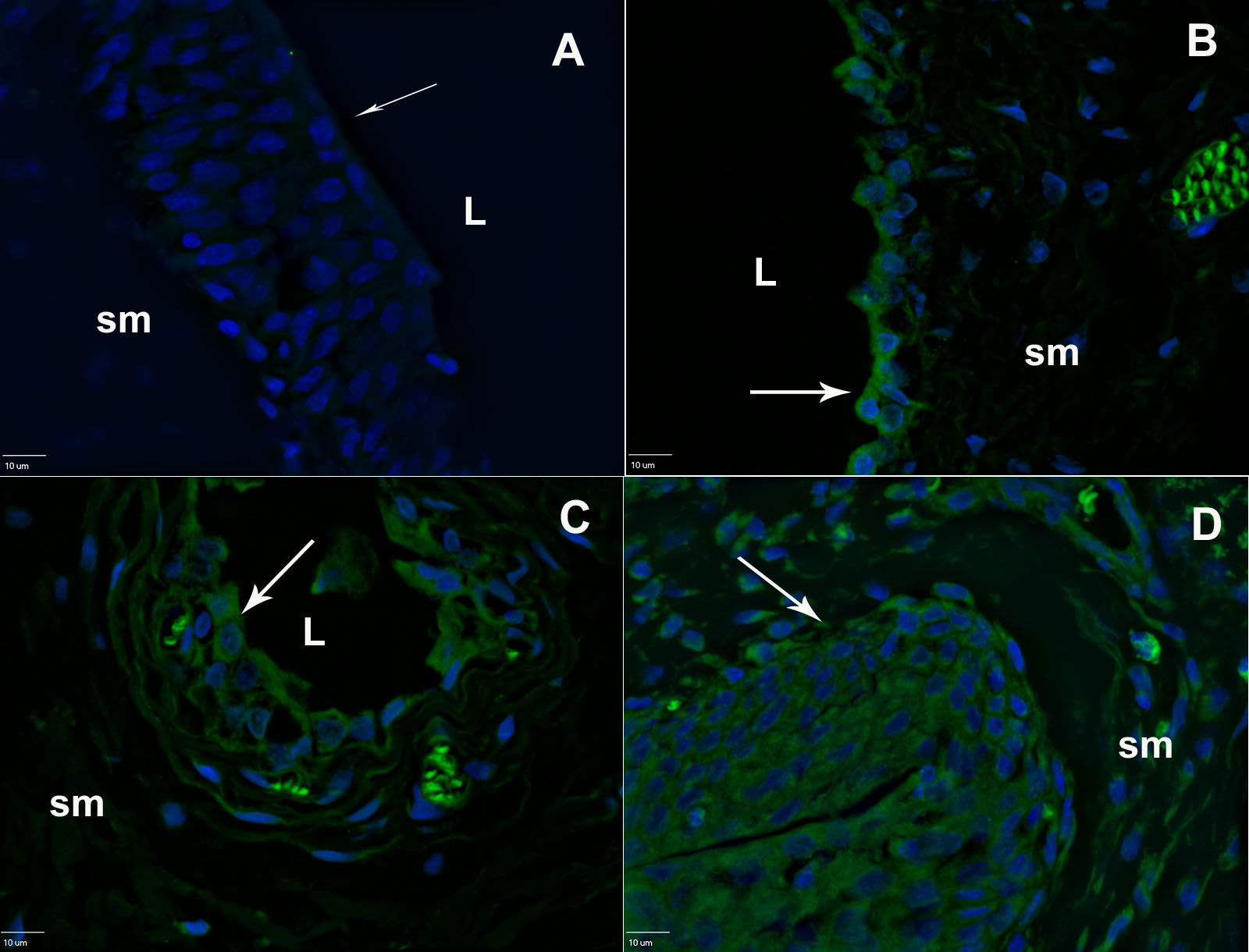

Supplement: Additional file 2 — Immunohistochemical detection of intact filamin A in the bladder tissue of F344 rats inoculated with sterile 10B broth or 109 CFU of U. parvum. Representative bladder tissue sections from isotype primary antibody control (A), sham inoculated control (B), asymptomatic UTI (C), and struvite (D) groups demonstrating the distribution of intact filamin A (green). Nuclei were stained with DAPI (blue). Images are 600× magnification, L = bladder lumen, SM = submucosa, and arrows are pointing to uroepithelium. [file 1471-2334-11-101-S2.JPEG]

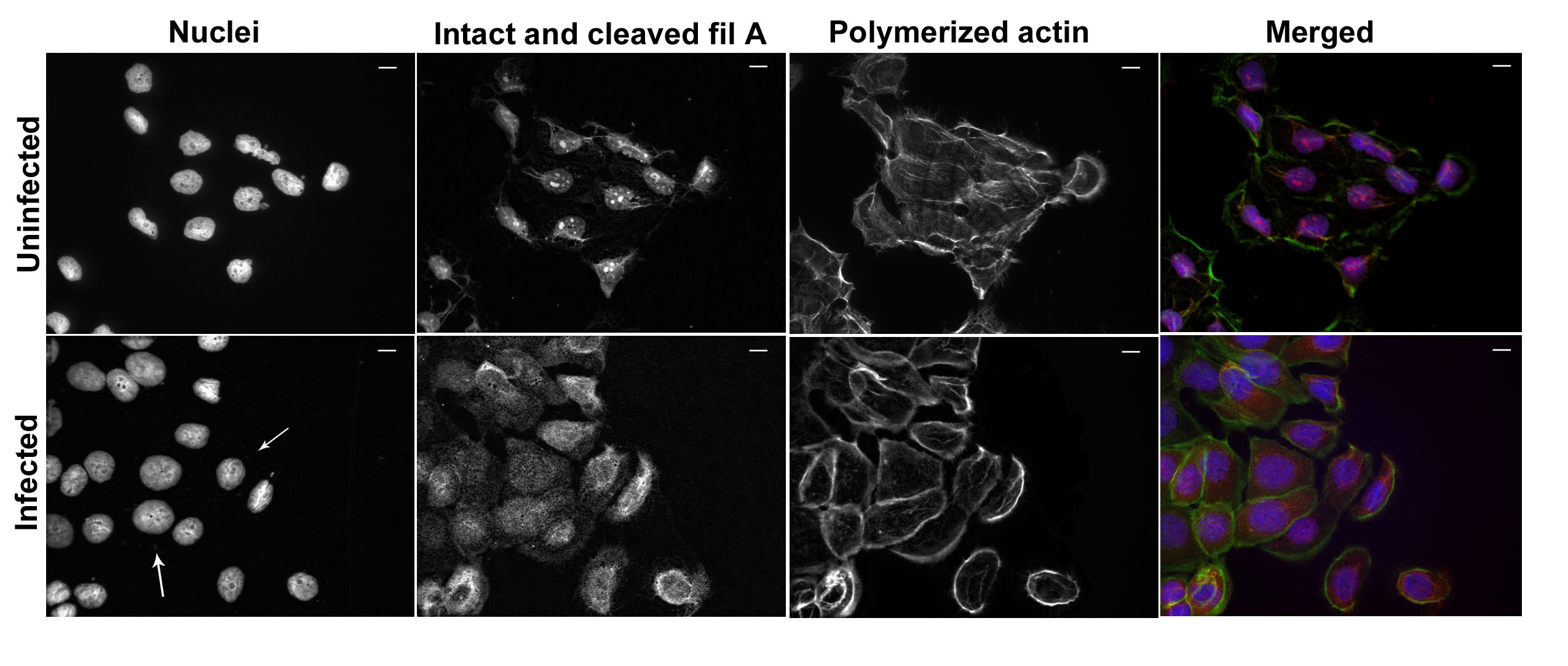

Supplement: Additional file 3 — Colocalization of filamin A with polymerized actin in uninfected and U. parvum infected BPH-1 cells. Representative images of cells examined 72 hours after inoculation with sterile 10B broth or 109 CFU of U. parvum. Nuclei were stained with DAPI (blue), white arrows are pointing to U. parvum colonies that were detected with DAPI staining. Filamin A (Fil A) was stained with rabbit- anti C terminal filamin A (red). Polymerized actin was stained with phalloidin Alexa-448 (green). All images were taken at 400× magnification and the scale bar is equal to 10 μm. [file 1471-2334-11-101-S3.JPEG]
